# Discovering Innate Driver Variants for Risk Assessment of Early Colorectal Cancer Metastasis

**DOI:** 10.3389/fonc.2022.898117

**Published:** 2022-06-20

**Authors:** Ruo-Fan Ding, Yun Zhang, Lv-Ying Wu, Pan You, Zan-Xi Fang, Zhi-Yuan Li, Zhong-Ying Zhang, Zhi-Liang Ji

**Affiliations:** ^1^ State Key Laboratory of Cellular Stress Biology, National Institute for Data Science in Health and Medicine, School of Life Sciences, Xiamen University, Xiamen, China; ^2^ Department of Clinical Laboratory, Xiamen Xianyue Hospital, Xiamen, China; ^3^ Department of Clinical Laboratory, Zhongshan Hospital , affiliated to Xiamen University, Xiamen, China

**Keywords:** colorectal cancer, metastasis, local invasion, driver variants, machine learning

## Abstract

Metastasis is the main fatal cause of colorectal cancer (CRC). Although enormous efforts have been made to date to identify biomarkers associated with metastasis, there is still a huge gap to translate these efforts into effective clinical applications due to the poor consistency of biomarkers in dealing with the genetic heterogeneity of CRCs. In this study, a small cohort of eight CRC patients was recruited, from whom we collected cancer, paracancer, and normal tissues simultaneously and performed whole-exome sequencing. Given the exomes, a novel statistical parameter LIP was introduced to quantitatively measure the local invasion power for every somatic and germline mutation, whereby we affirmed that the innate germline mutations instead of somatic mutations might serve as the major driving force in promoting local invasion. Furthermore, *via* bioinformatic analyses of big data derived from the public zone, we identified ten potential driver variants that likely urged the local invasion of tumor cells into nearby tissue. Of them, six corresponding genes were new to CRC metastasis. In addition, a metastasis resister variant was also identified. Based on these eleven variants, we constructed a logistic regression model for rapid risk assessment of early metastasis, which was also deployed as an online server, AmetaRisk (http://www.bio-add.org/AmetaRisk). In summary, we made a valuable attempt in this study to exome-wide explore the genetic driving force to local invasion, which provides new insights into the mechanistic understanding of metastasis. Furthermore, the risk assessment model can assist in prioritizing therapeutic regimens in clinics and discovering new drug targets, and thus substantially increase the survival rate of CRC patients.

## Introduction

Colorectal cancer (CRC) is one of the most frequent cancers worldwide and has the highest mortality after lung cancer (1, 2). The low survival rate and the high recurrence of CRC could be largely attributed to metastasis (3). About 20% of CRC patients already have metastases at diagnosis (4). Therefore, early assessment of metastasis risk can assist in prioritizing therapeutic regimen and thus substantially reduce the mortality of CRC patients.

Accumulating lines of evidence indicate that genetic factors may play a crucial role in CRC metastasis (5). However, CRC metastases are mechanistically heterogeneous, and the heterogeneity may answer for the poor prognosis in clinics. To date, the genomic basis of this variability has not been fully illustrated yet. With the goal of identifying driver genes/mutations in metastasis, previous works performed comparative lesion sequencing of matched primary versus metastatic CRC in cohorts of different size, race, age, and metastatic sites (4, 6–9). Some studies attempted to seek a high genomic concordance between primary and metastatic CRCs (7, 9–11), in which the concordant genomic biomarkers were thus taken as effective indicators for both diagnostic and prognostic implications of CRCs (6). These biomarkers, for example, BRAF mutations, were applied to assess mortality of metastatic CRC (12). A recent meta-analysis on 61 clinical studies and 3,565 metastatic CRCs concluded that four highly concordant gene biomarkers (KRAS, NRAS, BRAF, and PIC3KA) might drive the metastatic spread (6). However, due to the interference of “background noise” produced by extensive heterogeneity of the tumor cell variations, biomarker discordance was also often observed. For instance, the discordance rates of KRAS mutations between primary CRC and its metastases could be as high as 22% (13). PIK3CA demonstrated a 6.8-fold higher odds of discordance between the primary and the metastatic sites (14). In addition, it was reported that 65% of somatic mutations originated from a common progenitor, in which 15% were tumor-specific and 19% were metastasis-specific (15). Alternatively, some studies paid more attention to the metastasis-specific alterations (5, 16). A previous study suggested that targeted therapy of colorectal liver metastases would be more effective on the basis of the genetic properties of metastasis rather than those of the primary tumor since there was a significant genetic difference (17). However, a phylogenetic analysis of pancancer metastases manifested that many genetic biomarkers or driver genes were common to all CRC metastases, and the driver gene mutations not shared by all metastases were unlikely to have functional consequences (8). After all, these efforts discovered a bundle of potential metastasis-associated genes that were recurrently mutated at the metastatic sites, including APC, TP53, KRAS, PIK3CA, and SMAD4 (**Table 1**). It should be noted that many of the metastasis-associated genes are also involved in CRC origin and progress (4).

**Table 1:** Summary table of the CRC metastasis-associated genes *via* literature research.

Gene	Description	Association
NRAS	N-RAS oncogene encoding a membrane protein	RAS signaling has been involved in the initiation of epithelial-to-mesenchymal transition (EMT) in CRC leading to tumor spreading (18).
BRAF	Encodes a protein belonging to the RAF family of serine/threonine protein kinases	BRAF mutation was related to CRC metastasis and distant metastasis in an Asian population (18).
KRAS	Kirsten RAS oncogene homolog from the mammalian RAS gene family	KRAS mutation was associated with lymphatic and distant metastases in CRC patients (19).
PIK3CA	Phosphatidylinositol 3-kinase	PIK3CA mutation was associated with lung metastases in metastatic colorectal cancer (20).
NF1	Negative regulator of the RAS signal transduction pathway	Dysregulated NF1 expression promotes cell invasion, proliferation, and tumorigenesis (21).
PTEN	Encodes phosphatidylinositol-3,4,5-trisphosphate 3-phosphatase	Loss of PTEN expression contribute to CRC development and is associated with the migration aggressive capacity (22).
APC	Encodes a tumor suppressor protein that acts as an antagonist of the Wnt signaling pathway	APC mutation caused intestinal adenomas and combination with Trp53R270H mutation or TGFBR2 deletion induced submucosal invasion (23).
TP53	Encodes a tumor suppressor protein containing transcriptional activation, DNA binding, and oligomerization domains	Combined inactivation of Mir34a and TP53 promotes azoxymethane-induced colorectal carcinogenesis and tumor progression and metastasis by increasing levels of IL6R and PAI1 (24).
SMAD4	Encodes a member of the SMAD family of signal transduction proteins acts as a tumor suppressor and inhibits epithelial cell proliferation	Activation of BMP signaling in SMAD4-negative cells altered protein and messenger RNA levels of markers of epithelial–mesenchymal transition and increased cell migration, invasion, and formation of invadopodia (25).
POLE	Encodes the catalytic subunit of DNA polymerase epsilon	POLE‐mutated CRCs arose in the transverse colon and rectum, and showed increased tumor‐infiltrating lymphocytes and immune cells at the tumor–stromal interface (26).
RHBDD1	Rhomboid Domain Containing 1	RHBDD1 regulated ser552 and ser675 phosphorylation of β-catenin to activate the Wnt signaling pathway resulted in the recovery of signaling pathway activity, migration, and invasion in CRC cells (27).
RNF183	Ring Finger Protein 183	RNF183 promotes proliferation and metastasis of CRC cells *via* activation of NF-κB-IL-8 axis (28).
LUZP1^*^	Encodes a protein that contains a leucine zipper motif	Expression of LUZP1 was specifically downregulated for liver metastasis of colon carcinoma (29).
ARHGEF17^*^	Rho Guanine Nucleotide Exchange Factor 17	ARHGEF17 was involved in Phospholipase C signaling, which contributed to the lung metastasis from colon cancer (30).
CCDC78^*^	Protein coding gene whose function unknown	CCDC78 gene silencing significantly suppressed the viability, migration, and invasion of colon cancer cells (31).
LBX2^*^	Putative transcription factor	LBX2 was correlated with advanced tumor stage (III or IV), vascular invasion, and lymphatic invasion in colorectal cancer (32).
WFDC10B^*^	Encodes a member of the WAP-type four-disulfide core (WFDC) domain family	Expression of WFDC10B significantly upregulated in the hepatic metastasis of colon carcinoma (33).
PLA2G4B^*^	Encodes a member of the cytosolic phospholipase A2 protein family	High expression of PLA2G4B can accelerate decomposition of cell membrane phospholipid proteins, enhance cellular membrane fluidity, then increase cell adhesion and migration (34, 35).

*Susceptible genes identified in this study.

In recent years, several prediction models were developed for tumor metastasis assessment. Some used conventional clinical pathological characteristics, such as age, race, gender, tumor site, and tumor size, to establish the Cox regression models (or the proportional hazards models) to assess metastasis and survival outcomes for CRC patients (18–20). Some applied nomograms to perform metastasis assessment on the basis of radiomics signatures (21–24). For instance, imaging descriptors derived from computed tomography (CT) were used as prognostic or predictive biomarkers for metastasis (25). With the widespread application of high-throughput sequencing technology, some research groups also mined multiple omics data for metastasis assessment. For examples, Kandimalla et al. constructed an 8-gene classifier based on gene expression profiles to predict lymph node metastasis in T1 CRC patients (26). Ozawa et al. used five microRNA signatures to predict lymph node invasion in T1 CRC cancers (27). Regretfully, despite the enormous efforts that have been made to identify biomarkers and build prediction models for CRC metastasis risk assessment, there is still a huge gap to translate these efforts into clinical applications due to the problem of poor consistency (28, 29). In particular, they are powerless on risk assessment of early CRC metastasis.

Tumor metastasis is an invasive action of tumor cells, which refers to the process of tumor cells spread to other parts of the body. In principle, metastasis usually progresses in four steps: local invasion, intravasation into the blood circulation system, extravasation into the surrounding tissues, and colonization and proliferation in new locations (30). Local invasion of tumor cells is the initial step of almost all types of metastases (31). Before the tumor cells detach from the primary lesion, they proliferate and spread to nearby tissues, and communicate with adjacent cells in response to the microenvironment changes (32). Therefore, instead of identifying concordant gene markers between the start point (primary tumor) and the end point (metastatic tumor), exploring the driving genetic force at the initial step (local invasion) may capture the true signals of early metastasis. Unfortunately, few studies have been ever undertaken to date to identify local invasion-associated genes in malignant cancers.

In this work, we attempted to mine driver genes/mutations in early CRC metastasis. For this purpose, we elaborately designed an experiment to profile genomic alternation landscapes of cancer, paracancer, and normal tissues simultaneously in a CRC cohort. Upon the genomic mutation profiles, a new statistical parameter was introduced to quantitatively evaluate the contribution of every mutation to local invasion. Subsequently, we identified metastasis driver mutations *via* mining multiple omics data derived from different CRC sources. Lastly, we developed a machine learning model for rapid assessment of early CRC metastasis.

## Data and Methods

### The CRC Cohort

This study was approved by the Ethics Committee of the Xiamen Xianyue Hospital and was performed in accordance with the Helsinki Declaration. All patients provided written informed consent prior to inclusion in the study. A total of eight CRC inpatients from the Zhongshan Hospital, affiliated to Xiamen University, Fujian Province, China were recruited in this study. They were selected from more than 248 CRC inpatients on the basis of the following criteria: (1) the patients have no blood kinship by medical background review; (2) the patients were diagnosed with rectal differentiated adenocarcinoma of stage II or III; and (3) the patients received a similar chemotherapy regimen and the prognoses were benign. These eight patients were further divided into two groups: the NM group of four patients who had no metastasis till surgery excision, and the LM group of four patients who had local lymphatic metastasis but no distal metastasis. The medical details of the patients are briefly summarized in **Table 2**.

**Table 2:** Detailed information of the CRC patients.

Sample ID	Gender	Age	Pathological Diagnosis	Medication	Prognosis	10-month prognosis
N1	Female	51	RAMD, T4aN0M0, IIB	Oxaliplatin, Tegafur	Benign	Benign
N2	Male	59	RAMD, pT4aN0M0, IIB	Oxaliplatin, Capecitabine	Benign	Benign
N3	Male	53	RAMD, T4aN0M0, IIB	Oxaliplatin, Capecitabine	Benign	Benign
N4	Male	60	RAMD, pT4aN0M0, IIB	Xeloda	Benign	Benign
L1	Male	54	RAMD, pT4aN1M0, IIIB	Oxaliplatin, Capecitabine	Benign	Benign
L2	Female	48	RAMD, pT4aN1aM0, IIIB	Oxaliplatin, Capecitabine	Benign	Not Available
L3	Male	47	RAMD, T4aN2M0, IIIC	Oxaliplatin, Capecitabine	Benign	Benign
L4	Male	54	RAMD, pT4aN2bM0, IIIC	Oxaliplatin, Capecitabine	Benign	Liver and lung metastases

### Experiment Design and Sample Collection

**Figure 1:**
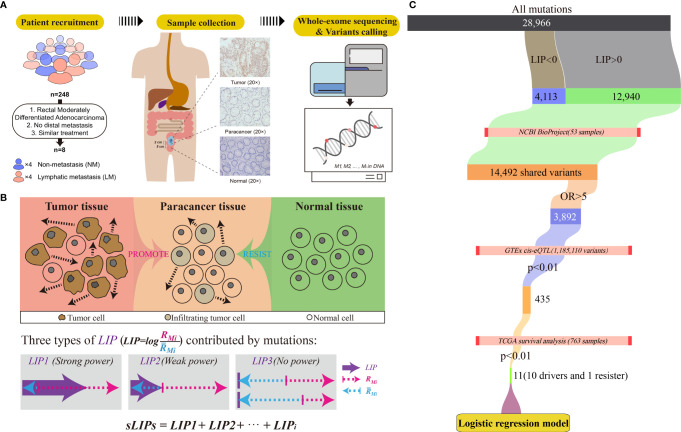
Workflow of the study. **(A)** Criteria and procedures of the sample collection and tissue selection. **(B)** Schematic diagram of the LIP calculation. ${\bar{\text{R}}}_{\text{Mi}}$ Rmi stands for the invasion promotion rate, and ${\bar{\text{R}}}_{\text{Mi}}$ stands for the invasion resistance rate. **(C)** Schematic diagram of identification of germline driver mutations for early risk assessment of CRC metastasis.

For every patient in the cohort, three tissue samples were collected from the tumor removal surgery under authorization in advance: the tumor sample was collected at the near edge of the tumor, and the paracancer and normal samples were taken 2 cm and 5 cm away from the tumor, respectively (**Figure 1A**). Overall, 24 tissue samples of eight patients were collected. The pathological status of tissue samples was determined by standard immunohistochemistry (IHC) examination. The tissue samples were frozen in liquid nitrogen soon after the surgical excision and kept at −80°C for long-term storage.

### Mutation Profiling With the Whole-Exome Sequencing

The genomic DNAs of tissue samples were extracted using the EZ-10 Spin Column Blood Genomic DNA Purification Kit (Sangon Biotech Co, Ltd., Shanghai, China). The DNA concentration was measured by a Qubit Fluorometer and diluted to 50–300 ng/µl. For each sample, 3–5 µg of DNA was applied for quality control, and its integrity was checked by the agarose electrophoresis. The whole exome was captured using the MGIEasy Exome Library Prep Kit (BGI, Shenzhen, China) and the library for sequencing was prepared according to the manufacturer’s instruction. The whole-exome sequencing (WES) was performed by the Beijing Genome Institute (BGI, Shenzhen, China) using the BGISEQ-500 platform in a 100-base pair (bp) paired-end mode.

### Exome Data Preprocessing, Variants Calling, and Variant Annotation

Before variant calling, quality control was conducted to the sequencing raw data using Trimmomatic (v.0.39; parameters: LEADING=20, TRAILING=20, SLIDINGWINDOW=5:20, MINLEN=80) (51). The clean reads were mapped to the human reference genome (GRCh38.p12) using the Burrows-Wheeler Aligner (BWA, v.0.7.17; parameters: mem -t 4 -M -R) (52). We used the Genome Analysis Toolkit (GATK, v.4.1.1.0) (53) and the Samtools (v.1.9) (54) for basic processing, duplicate marking, and base quality scores recalibrating (BQSR). Variant calling for germline mutations and somatic mutations was conducted using GATK HaplotypeCaller and Mutect2, respectively. The variants were further annotated with the ANNOVAR (v2019Oct24) (55).

### Estimation of Tissue Purity and Ploidy

For every tumor and paracancer samples, the tissue purity and ploidy were estimated on the basis of genome-wide somatic mutation profiles with Sclust (v.1.1, -t tumor.bam -n normal.bam -rc -minp 2 -maxp 3.5) (56), taking the corresponding normal tissue as the reference.

### Calculation of Local Invasion Power

Every mutation likely plays dilemmatic roles in metastasis, promotion, or resistance. For a gene mutation, M*_i_* if the driving potential outmatches the resisting potential, M*_i_* is considered as the driver mutation to metastasis; otherwise, M*_i_* is the resister mutation. To measure the summarized potential of M*_i_* to local invasion, a novel parameter, namely, local invasion power (LIP), was introduced:


(1) $${\text{LIP}}_{i}=\;\log\frac{{\text{R}}_{\text{Mi}}}{{\bar{\text{R}}}_{\text{Mi}}}$$

where R_Mi_ and ${\bar{\text{R}}}_{\text{Mi}}$ stand for the invasion promotion rate and the invasion resistance rate, respectively. The logarithm (log) took 2 as the base. R_Mi_ and ${\bar{\text{R}}}_{\text{Mi}}$ were calculated by:


(2) $${\text{R}}_{\text{Mi}}{\text{= V}}_{\text{MPi}}{\text{ / V}}_{\text{MTi}}$$

(3) $${\bar{\text{R}}}_{\text{Mi}}{\text{= V}}_{\text{MTi}}{\text{ / V}}_{\text{MNi}}$$

where V_MTi_ , V_MPi_ and V_MNi_ stand for the variant allele fraction (VAF) of variant M*_i_* in tumor, paracancer, and normal tissues, respectively. They were determined by dividing reads of alternate allele M*_i_* by total reads at this locus and further normalized by all reads count. LIP > 0 indicated that the variant M*_i_* was prone to promoting invasion than resistance. A larger LIP suggested that the mutation had more power to drive local invasion.

Moreover, we assume that the tumor invasion is the accumulated consequence of all mutations. Some mutations likely promote tumor cells invading into nearby tissue (paracancer tissue), while some intend to resist the invasion. If the overall promotion effects at the paracancer tissue overwhelm the resistance effects, local invasion is prone to progress; otherwise, invasion unlikely happens (**Figure 1B**). We also assume that the impact of mutations on the invasion is linear. Accordingly, the invasion risk of whole mutation profiles can be simply determined by calculating the summation of LIPs (sLIPs):


(4) $$sLIPs=\sum_{i=1}^{n}LIP_{i}$$

where *n* is the number of mutations involved in the analysis.

### Identification of Metastasis Driver Variants

We identified potential metastasis driver variants by cascade bioinformatic analyses (**Figure 1C**): (1) By setting a threshold of LIP > 0, we obtained the list of invasion-promoting variants that were determined upon the CRC cohort of this study. (2) We estimated metastasis-variant association for the invasion-promoting variants by conducting the odds ratio (OR) analysis on the basis of external CRC datasets collected from the NCBI BioProject. The datasets were chosen by multiple criteria: (i) the CRC cohort consisted of both metastasis and non-metastasis cases; (ii) the mutation profiles were determined by WES; and (iii) the clinical information such as metastasis status was acquirable. Results show that three datasets met all criteria and were included in the OR analysis: PRJNA494574 (10 samples) (57), PRJNA514428 (24 samples) (58), and PRJNA246044 (19 samples) (41). Of these 53 CRC samples, 28 had either lymphatic metastasis or distal metastasis, and the remaining 25 did not observe metastasis by the time of experiment. The raw sequencing data of these datasets were downloaded and preprocessed, and germline variants were called, following exactly the same operations as described above. For OR analysis, the contingency table was constructed and the OR values for every selected variants were calculated by:


(5) $$OR=\frac{M_{m}N_{n}}{M_{n}N_{m}}$$

where M*_m_* and M*_n_* stand for the number of mutations and non-mutations (the wild type) at the selected allele in the metastasis group, respectively. N*_m_* and N*_n_* stand for the number of mutations and non-mutations at the selected allele in the non-metastasis group, respectively. As a result, a list of metastasis-associated variants with OR >5 was determined. (3) The genetic predisposition of metastasis-associated variants to patient survival was examined. For this, the gene expression level interfered by mutation was first determined according to the expression quantitative trait loci (eQTL) information derived from the Genotype-Tissue Expression (GTEx) (60). Only the significant (*p <*0.01) variants to either sigmoid or transverse colons were included in the analysis, which were 1,185,110 variants in the GTEx. Having the information of mutations on gene expression levels, we then performed survival analysis subject to high or low gene expression on the basis of 763 CRC patients (including 571 colon and 192 rectum patients) from The Cancer Genome Atlas (TCGA) using the R packages survival (v3.2-3) and survminer (v0.4.8) with default parameters. As a result, we screened out eleven effective variants that could change the host gene expressions and subsequently affect the survival of patients (*p* < 0.01). These eleven effective variants included ten potential metastasis driver variants that may reduce the survival rate of CRC patients and one resister variant on the opposite.

### Logistic Regression Model for Metastatic Risk Assessment

To aid risk assessment of early metastasis, we built a determinant classifier. The core component of classifier was a logistic regression model. The model was constructed on the basis of four exome datasets of this study and three independent CRC cohorts (NCBI BioProject: PRJNA514428, PRJNA246044, and PRJNA494574), covering a total 61 CRC patients. The datasets were split into a training set and a testing set in a combinational way (**Table 3**). The training set consisted of any three of four exome datasets, which were used for model construction and internal evaluation; the remaining dataset was taken as the testing set for external evaluation, which was independent of model construction.

**Table 3:** Model construction and performance evaluation.

Dataset		Internal evaluation				External evaluation			
Training set	Testing set	AUC	Accuracy	Sensitivity	Specificity	AUC	Accuracy	Sensitivity	Specificity
PRJNA246044, PRJNA494574, and this study	PRJNA514428	0.772	0.729	0.727	0.730	0.675	0.833	0.905	0.333
PRJNA514428, PRJNA494574, and this study	PRJNA246044	0.834	0.738	0.750	0.700	0.793	0.842	0.736	0.600
PRJNA514428, PRJNA246044, and this study	PRJNA494574	0.932	0.882	0.840	0.923	0.667	0.700	0.714	0.667
PRJNA514428, PRJNA246044, PRJNA494574	This study	0.803	0.804	0.760	0.846	0.700	0.690	0.714	0.667
**Average**		0.835	0.788	0.769	0.800	0.709	0.766	0.767	0.567

The model took the mutation profiles of eleven metastasis-associated driver variants identified in this study as the input, and output the estimated probability of metastatic risk. In model construction, the input genetic mutation profile was converted into a one-dimension 11-feature binary vector **V**, corresponding to the eleven metastasis-associated variants, in which carrying the mutation was defined as 1, otherwise 0.


(6) $$\text{V}=\left(V_{1},\;V_{2},\ldots ,V_{11}\right)$$

Meanwhile, a weighted vector **L** was prepared for **V** ,which contained the average LIPs of the eleven metastasis-associated variants determined on the basis of the training dataset.


(7) $$\text{L}=\left(LIP_{1},\;LIP_{2},\ldots ,LIP_{11}\right)$$

Accordingly, we calculated the dot product of **V** and **L (V·L)** as the accumulated driving force of metastasis contributed by the eleven variants for the patient. For the metastasis issue (*y* = 1) . the probability of occurrence P (*y* = 1) can then be determined by the logistic regression:


(8) $$\begin{array}{l} P\left(y\right)=\frac{1}{1+exp\left(\sum_{i=1}^{11}-w_{i}V_{i}L_{i}-b\right)} \end{array}$$

where *w_i_* is the regression coefficient for the variant and *b* is the intercept. The regression coefficient *w_i_* and intercept *b* were estimated using the Maximum Likelihood Estimation (MLE) with the glm function of the R package stats (v3.6.0).

The model performance was evaluated by the conventional parameters of accuracy, sensitivity, and specificity, which were calculated with the R function confusionMatrix from the package Caret (v6.0-86) as follows:


(9) $$Accuracy=\frac{TP+TN}{P+N}$$

(10) $$Sensitivity=\frac{TP}{TP+FN}$$

(11) $$Specificity=\frac{TN}{TN+FP}$$

where *P* and *N* stand for the positives and the negatives, respectively. The values of *TP* (true positives), *TN* (true negatives), *FN* (false negatives), and *FP* (false positives) were calculated on the basis of the confusion matrices of the classification model. The area under the receiver operating characteristic curve (AUC) was also determined with the R package pROC (v1.16.2). For evaluation of all models, the leave-one-out cross-validation (LOOCV) strategy was applied to attain unbiased estimation of training. For this purpose, the training dataset was divided 51-fold (corresponding to 51 patients), of which 50 were used for model construction and the remaining one was used for internal evaluation. The LOOCV process was repeated 51 times, and the average parameters were used to evaluate the model performance of the training set.

## Results

### Determination of Local Invasion Power Based on Mutation Profiling

**Figure 2:**
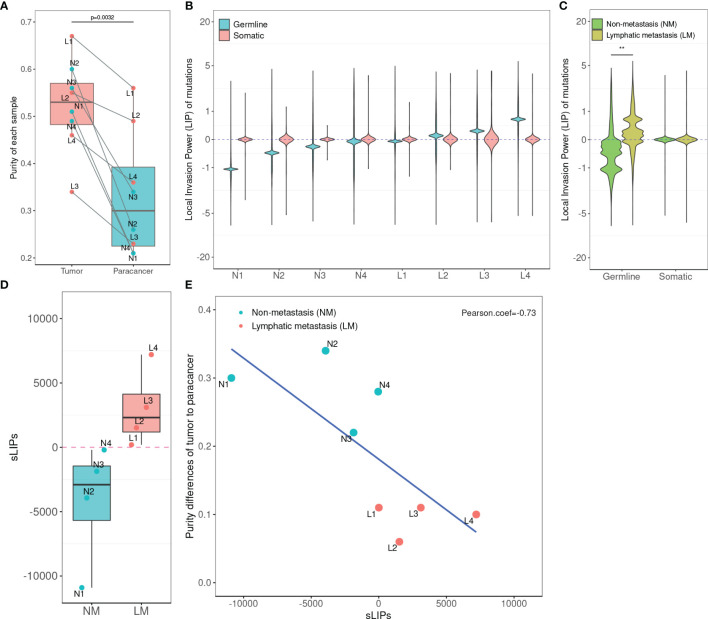
Statistics of tumor purity in the 8-patient CRC cohort and the correlation with LIPs. **(A)** Purity of tumor and paracancer. The one-sided paired t-test was used to determine the difference between two groups. **(B)** Distribution of LIPs. The blue stands for the distribution determined on germline variants and the red stands for that on somatic variants. The *x*-axis is the subject name and the *y*-axis is the value of LIP. **(C)** The superimposed LIP distribution. Green stands for the non-metastasis group (NM) and yellow stands for the lymphatic metastasis group (LM). The Wilcoxon rank-sum test was used to determine the difference between the two groups. **(D)** The boxplot of sLIP comparison between the NM group and the LM group. **(E)** The Pearson correlation analysis between the sLIP and the tumor-to-paracancer purity change. **p < 0.01.

After quality control, WES of the 8-patient CRC cohort (24 tissue samples) produced an average on-target coverage of about 197×, indicating that the sequencing was substantially deep enough for reliable variant calling. Using the matched normal samples as reference, we determined the purities of tumor and paracancer tissue for every patient based on the genome-wide somatic mutation profiles. On average, the purity of tumor samples was significantly higher than that of matched paracancer samples (one-tailed paired *t*-test, *p* = 7.97e-4). The average purity of tumors and paracancer tissues was 0.52 and 0.33, respectively (**Figure 2A**). This result manifests that the genetic basis of paracancer tissues has changed significantly from that of normal tissues, though the cells have not yet exhibited a morphologically visible difference.

In the cohort, a total of 12,880 distinct and nonsynonymous somatic mutations were called, including 5,069 SNVs (single-nucleotide variants) and 8,275 indels (inserts and deletions). For every mutation, we calculated the LIP; meanwhile, we determined the summation of all mutation LIPs (namely, sLIP) for every cohort member. Regretfully, both the LIP distribution and sLIPs were unable to differentiate the lymphatic metastasis group (LM) from the non-metastasis group (NM) (**Figures 2B, C**). This finding challenges somatic mutations as the major driving force to local invasion.

Alternatively, we turned to seek clues from the germline mutations. Overall, 28,966 nonredundant nonsynonymous germline mutations were called in the cohort, including 619 nonsense SNVs, 25,169 missense SNVs, and 3,178 indels. In the same way, we calculated LIPs for every potential effective germline mutations and sLIPs for every cohort member. As illustrated in **Figure 2B**, the cohort members had different LIP distributions but a similar style, which the majority of LIPs valued at a narrow range. The different LIP distributions indicated different risk levels of local invasion; the larger LIP, the riskier. In general, the LM members had significantly larger LIPs than NM members (**Figure 2C**). The LM members all had a sLIP > 0; in contrast, the NM members all had a sLIP < 0. Furthermore, the sLIP value was positively correlated with the metastatic status of CRC (**Figure 2D**). For instance, patients L1 and L2 of the LM group were diagnosed as early stage of local lymphatic metastasis (N1), which had significantly lower sLIP values compared to that of patients L3 and L4 of metastasis stage N2. In particular, patient L4 who was diagnosed with liver and lung metastases 10 months after surgery had the largest sLIP value (7,204.88) in the cohort. In addition, we conducted a correlation analysis between the sLIP value and the tumor-to-paracancer purity change for every patient involved. A significant negative correlation was observed (Pearson coefficient = −0.732 and *p* = 0.039) (**Figure 2E**). These results suggest that the LIP value could properly reflect the contribution of mutation to the metastasis, and sLIP could serve as a good indicator of metastasis status.

### Identification of Metastasis Driver Variants

**Figure 3:**
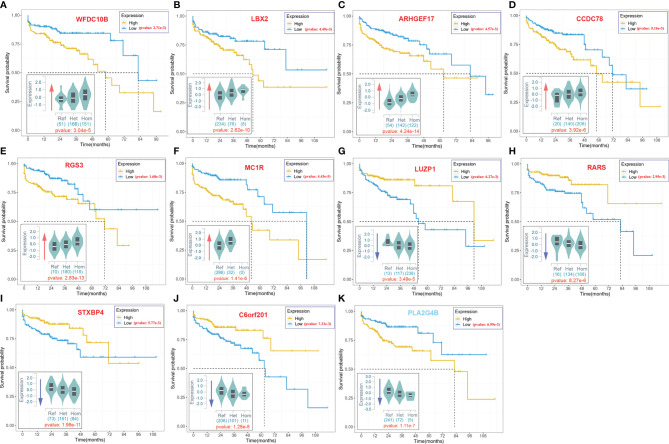
The 10-year Kaplan–Meier survival analysis for ten metastasis driver mutations (gene symbol in red) and one resister mutation (gene symbol in blue).. The violin figure at the bottom left corner in each subgraph stands for mutation effect on parental gene expression based on the cis-expression quantitative trait locus (cis-eQTL) analysis of the GTEx. The *x*-axis stands for the genotype of allele, and the *y*-axis stands for the normalized expression. The red arrow indicates upregulation of the host gene expression by the mutation. The blue arrow indicates downregulation of the host gene expression by the mutation. The number under the violin figure stands for the number of corresponding genotype samples in total 318 samples involved in the cis-eQTL analysis. The significance of analysis is labeled in red.

As illustrated in **Figure 2C**, some variants (LIP > 0) contributed positively to metastasis. These variants were the potential driver variants that, to some extent, determined the incidence of metastasis. Hence, to identify the metastasis driver mutations consensus to most CRC cases, we conducted three-step bioinformatic analyses (**Figure 1C**): (1) From the 8-patient cohort of this study, we extracted 13,089 distinct variants that promoted the metastasis (mean value of LIP > 0), of which 186 had mean LIP > 1. (2) Then, we affirmed the mutation-metastasis association by including 53 additional CRC cases (28 metastasis and 25 non-metastasis) from three independent cohort studies. Overall, 2,751 variants were found to be highly associated with metastasis with OR > 5, and 16 were also in the list of high metastasis-promoted variants. (3) Lastly, we examined the impact of mutations on gene expressions and thereby the penetration to metastasis *via* mining big data from the GTEx and the TCGA (763 CRC patients). In the end, we obtained ten potential driver variants to metastasis. These variants can enhance (six variants) or suppress (four variant) their parental gene expression, and all would consequently shorten the lifetime of half survivals for an average of 31.5 months (**Figure 3**). There were nine SNVs (WFDC10B rs232729, LBX2 rs17009998, CCDC78 rs2071950, RGS3 rs10817493, MC1R rs885479, LUZP1 rs477830, RARS rs244903, STXBP4 rs1156287, and C6orf201 rs619483) and one insertion (ARHGEF17 rs113363731) (**Table 4**). Of these ten genes, five genes (WFDC10B, LBX2, CCDC78, LUZP1, and ARHGEF17) were previously reported to participate in nearby cell invasion, and lymphatic and distant CRC metastases (**Table 1**). Three genes (RARS, MC1R, and RGS3) were involved in tumor metastasis other than CRC (**Table 4**). For the remaining two genes (STXBP4 and C6orf201), their connections with metastasis have not been reported yet. However, STXBP4 can facilitate cell directional migration (61) and C6orf201 is related to the mesodermal commitment pathway (62). It is noteworthy that all these variants were common variants in the global population, owning an estimated allele frequency >10% in the ExAC database (63). Six of them even had a high frequency >60% of population. All these results suggested that the ten metastasis driver variants/genes had a substantial population basis and could serve as good biomarkers in monitoring CRC metastasis. Other than the ten metastasis driver variants, we also detected one metastasis resister variant: PLA2G4B rs3816533 (**Table 4**). This variant was highly associated with (OR > 5) and resistant (LIP < −1) to CRC metastasis (**Figure 3K**). PLA2G4B encodes phospholipase 2A. The high expression of phospholipase 2A may accelerate decomposition of cell membrane phospholipid proteins, which enhance cellular membrane fluidity, a critical modulator of cell adhesion and migration (49). The change in cellular membrane fluidity may increase metastatic capacity (50). Notably, PLA2G4B was reported to be specifically upregulated in liver metastasis of colon carcinoma (44).

**Table 4:** Detailed information of metastasis driver/resister mutations.

dbSNP ID	Ref	Alt	Gene	Class*	Odds ratio	*p* (cis-eQTL)	*p* (Survival analysis)	Association with metastasis
rs232729	A	G	WFDC10B	MP	5.06	1.42E-09	2.71E-03	Expression of WFDC10B significantly upregulated in the hepatic metastasis of colon carcinoma (33)
rs17009998	G	A	LBX2	MP	12.93	2.53E-23	4.49E-03	LBX2 was correlated with advanced tumor stage (III or IV), vascular invasion, and lymphatic invasion in colorectal cancer (32)
rs2071950	A	G	CCDC78	MP	+∞	1.98E-11	5.16E-03	CCDC78 gene silencing significantly suppressed the viability, migration, and invasion of colon cancer cells (31).
rs477830	C	T	LUZP1	MP	+∞	3.49E-05	6.27E-03	Expression of LUZP1 was specifically downregulated for liver metastasis of colon carcinoma (29).
rs113363731	–	CTC	ARHGEF17	MP	+∞	9.55E-06	4.57E-03	Mutations on ARHGEF17 contributed to the lung metastasis from colon cancer (30).
rs244903	G	A	RARS	MP	9.05	2.83E-13	2.95E-03	RARS encodes the arginyl-tRNA synthetases involved in oral cancer cell invasiveness (61).
rs885479	G	A	MC1R	MP	9.36	1.41E-06	4.43E-05	MC1R is melanocortin 1 receptor gene directly connected with activation of cell division and metastasis in malignant melanoma (62).
rs10817493	C	G	RGS3	MP	+∞	8.27E-06	1.68E-03	Higher expression of RGS3 was associated with a larger tumor size, lymph node metastasis, and local invasion in gastric cancer (63).
rs1156287	G	A	STXBP4	MP	+∞	3.92E-06	5.77E-03	STXBP4 can facilitate cell directional migration, which plays a role in tumor metastasis with an unknown mechanism (64).
rs619483	G	C	C6orf201	MP	5.52	1.28E-08	7.33E-03	C6orf201 is related to the mesodermal commitment pathway (65).
rs3816533	C	T	PLA2G4B	MR	5.59	1.11E-07	7.00E-3	High expression of PLA2G4B can accelerate decomposition of cell membrane phospholipid proteins, enhance cellular membrane fluidity, and then increase cell adhesion and migration (34, 35).

MP, metastasis promotion; MR, metastasis resistance.

### Logistic Regression Model for Early Metastatic Risk Assessment 

In this study, we were also motivated to construct a logistic regression model for CRC metastatic risk assessment. The model was built on the basis of the eleven strong metastasis-associated variants (ten drivers and one resister) instead of the whole germline mutation profiles that would be much more costly in practice. The model performance was internally evaluated in a manner of LOOCV, which obtained an average result: accuracy = 0.788, specificity = 0.800, sensitivity = 0.769, and AUC = 0.839. Additional external evaluation also achieved a fairly good performance: accuracy = 0.766, specificity = 0.567, sensitivity = 0.767, and AUC = 0.709. These results affirm that the model is substantially effective for early metastatic assessment.

**Figure 4:**
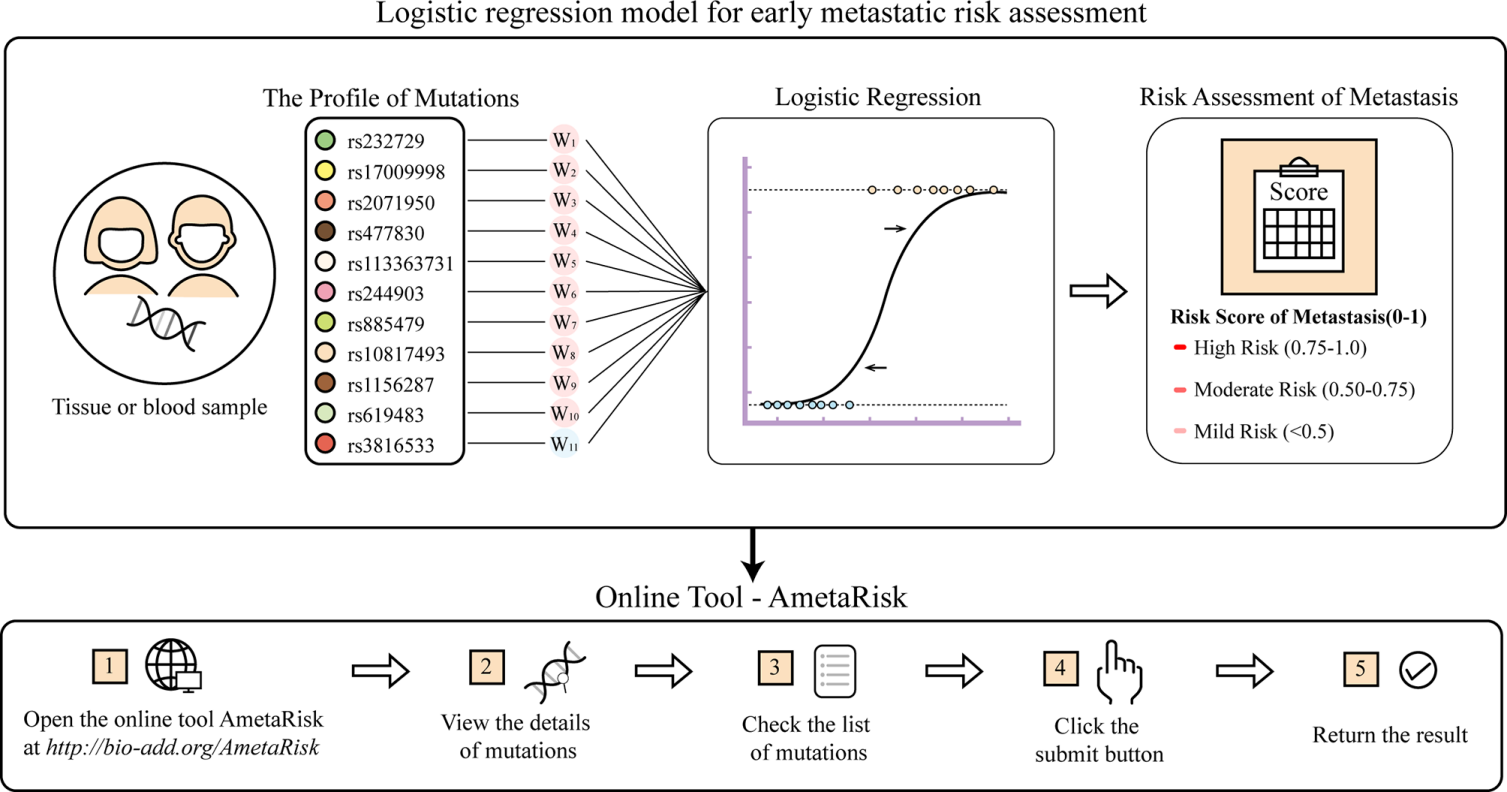
The AmetaRisk for interactive risk assessment of CRC metastasis.

For user convenience, we also deployed the model as an online tool, AmetaRisk, for interactive risk assessment of CRC metastasis, which can be freely accessible at http://www.bio-add.org/AmetaRisk. The AmetaRisk was built upon an architecture of Linux + Tomcat + JSP. To initiate the assessment, the user is required to check the status (yes or no) of eleven metastasis driver/resister variants detected in the tissue samples, which can be determined on tumor, paracancer tissue, or peripheral blood. Upon submission of variant status profile, the server will return a probability value of metastatic risk, ranging from 0 to 1.0 (**Figure 4**). According to the probability value, the metastatic risk can be categorized into three status: high risk (0.75–1.0), moderate risk (0.50–0.75), and mild risk (<0.5).

## Discussion

Early studies proposed that metastasis could progress *via* either a single lymphatic, hematogenous, or implantation route, or a combination of these (67). However, regardless of whichever route it may take, metastasis initiates through local invasion of tumor cells into nearby tissue (68, 69). The nearby tissue of cancer, or so-called paracancer tissue, is usually taken as normal control in many cases, but this study as well as several previous studies challenge this opinion. Although the cell morphology of paracancer tissue exhibits a pattern similar to that of normal tissue by IHC examination, the intrinsic genetic profile could have substantially changed. As determined by WES in this study, the mutation profiles of cancer, paracancer, and normal tissues were significantly different from each other. The cancer metastasis may have progressed already before it can be detected in the clinic. This provides us a good opportunity to investigate the genetic basis underlying metastasis.

In this study, we introduced a new statistical parameter, LIP, to characterize the contribution of genetic mutation to metastasis. The LIP value was calculated on the basis of relative variant allele frequency (VAF), a surrogate measure of the proportion of DNA molecules in the tissue specimen carrying the variant (70). The VAF to some extent reflects tumor heterogeneity, which also manifests the infiltration degree of tumor cells into paracancer tissue. Surprisingly, LIPs based on somatic mutation profiles failed to differentiate patients with local lymphatic metastasis from non-metastatic patients, which challenged somatic mutations as the major driving force to local invasion. Instead, LIPs based on germline mutation profiles could reflect the different pathological status of CRC patients. In particular, sLIPs were negatively correlated with the tumor purity change between cancer and paracancer tissues. All the results suggested sLIPs as a potential indicator for metastasis.

However, using sLIP value directly to assess metastatic risk may not be a good solution; many mutations actually contributed little to metastasis (71). The tremendous background mutations will overwhelm the true signals and thus lead to inaccurate metastatic risk assessment. Therefore, we mined the driver/resister variants that contributed most to the metastasis. Unlike previous studies that sought highly concordant genomic variants between primary and metastatic CRCs or metastasis-specific variants (6), we aimed at variants that drove local spread of tumor cells into paracancer tissue. For this purpose, we examined variant contribution to local invasion, variant-metastasis association, and variant impact on parental gene expression and patient survival. As a result, ten driver variants and one resister variant were identified. Similar attempts have not been reported previously. Upon these potential metastasis driver variants, we constructed a logistic regression model for early metastatic risk assessment and further deployed it as an online tool, AmetaRisk. To the best of our knowledge, this model would be the first model that makes quantitative risk assessment at the very early stage of metastasis before it actually occurs.

Last but not the least, unlike many studies that took somatic mutations as pathogenic drivers or biomarkers (72), this study was grounded on the hypothesis that germline mutations (inherited from the last generation) might be responsible for the “born-to-be-bad” characteristics of tumors, in which malignant progression has been determined long before visible invasion and metastasis were actually observed (73). Previous studies also identified several metastasis-associated germline variations, some of which were taken as prognosis markers of metastasis (74, 75). Many of them, such as KRAS, NRAS, BRAF, PIK3CA, and TP53, were also known as oncogenes. In **Table 1**, we summarized 18 potential metastasis driver genes/mutations identified to date. Comparing the gene list with the eleven driver/resister genes identified in this study, five genes (ARHGEF17, CCDC78, LBX2, LUZP1, and WFDC10B) were in common. These mutual genes have been reported to participate in the metastatic/invasive process. For instance, LBX2 is a transcription factor that is involved in diverse physiological processes and tumorigenesis. Upregulation of LBX2 in CRC may be associated with advanced tumor stage (III or IV), vascular invasion, and lymphatic invasion, which can be caused by the hypermethylation of LBX2 (59). ARHGEF17 (Rho Guanine Nucleotide Exchange Factor 17) contributes to the lung metastasis from colon cancer *via* participation in “phospholipase C signaling” (60).

We acknowledge that this study has several limitations. First of all, due to the difficulty of simultaneously collecting tumor, paracancer, and normal tissues, the study was demonstrated in a small cohort of eight patients. This may cause bias in LIP calculation and subsequent driver variant identification. Recently, WES studies of two larger CRC cohorts (146 patients and 618 patients, respectively) with a similar experiment design were reported (77, 78). Unfortunately, we were unable to acquire these datasets for mutation profile calling by all means. To complement the data gap, we strengthened the identification of metastasis driver variants by incorporating as many valid datasets derived from public databases such as NCBI, TCGA, and GTEx as possible. Moreover, this study focused on seeking inborn genetic bases of metastasis. However, both germline and somatic variants could together contribute to metastasis, as well as several other genetic features such as copy number variation (CNV) and structural variant (SV). Furthermore, this study used only eleven selected driver variants for metastatic risk assessment. The good part is that the variant selection largely reduces the tremendous background noise and enables achieving good performance under the circumstance of the small dataset (cohort). The bad part is that the simplified model may miss some useful information for a better performance. To improve this work, experimental validation of metastasis driver variants and involvement of more highly metastasis-associated variants are thus desired.

## Conclusion

In summary, we made a valuable attempt in this study to explore the genetic basis underlying CRC metastasis. Our efforts will provide new insights into the mechanistic understanding of early metastasis, as a complement to current metastasis hypotheses such as “seed and soil”, “big-bang”, and “tumor self-seeding”. Moreover, we constructed a machine learning model for metastatic risk assessment at the early stage of local invasion. This model and its online tool, AmetaRisk, provide a rapid and economic way to assist in prioritizing a precise therapeutic regimen in advance and increasing the survival rate of CRC patients in clinics.

## Data Availability Statement

The datasets presented in this study can be found in online repositories. The names of the repository/repositories and accession number(s) can be found at: https://ngdc.cncb.ac.cn/gvm/ (accession number: GVM000184).

## Ethics Statement

The studies involving human participants were reviewed and approved by the Ethics Committee of Xiamen Xianyue Hospital. The patients/participants provided their written informed consent to participate in this study.

## Author Contributions

Z-LJ and PY designed and supervised the study. PY, Z-XF, Z-YL, and Z-YZ collected the samples, performed the clinical diagnosis, and prepared the samples for sequencing. R-FD, YZ, and L-YW analyzed the data, and drafted and revised the manuscript. Z-LJ and PY commented on and revised the manuscript. All authors contributed to the article and approved the submitted version.

## Funding

This work was supported by the National Key Research & Developmental Program of China (2018YFC1003601).

## Conflict of Interest

The authors declare that the research was conducted in the absence of any commercial or financial relationships that could be construed as a potential conflict of interest

## Publisher’s Note

All claims expressed in this article are solely those of the authors and do not necessarily represent those of their affiliated organizations, or those of the publisher, the editors and the reviewers. Any product that may be evaluated in this article, or claim that may be made by its manufacturer, is not guaranteed or endorsed by the publisher.
